# Effect of the Structural Characteristics on Attachment-Detachment Mechanics of a Rigid-Flexible Coupling Adhesive Unit

**DOI:** 10.3390/biomimetics7030119

**Published:** 2022-08-26

**Authors:** Qijun Jiang, Liuwei Wang, Zhiyuan Weng, Zhouyi Wang, Zhendong Dai, Weidong Chen

**Affiliations:** 1 College of Mechanical and Electrical Engineering, Nanjing University of Aeronautics and Astronautics, Nanjing 210016, China; 2 College of Aerospace Engineering, Nanjing University of Aeronautics and Astronautics, Nanjing 210016, China; 3 Shenzhen Research Institute, Nanjing University of Aeronautics and Astronautics, Shenzhen 518063, China

**Keywords:** rigid-flexible coupling structure, bio-inspired adhesive unit, peeling, cohesive zone model (CZM), gecko

## Abstract

The terminal toes of adhesive animals are characterized by rigid-flexible coupling, and their structure–function relationship is an urgent problem to be solved in understanding bioinspired adhesive systems and the design of biomimetic adhesive units. In this paper, inspired by the rigid-flexible coupling adhesive system of the gecko toe, a rigid-flexible coupling adhesive unit was designed, the interface strength of the adhesives under different preloads was tested, and the model and analysis method of the compression and peeling process of the rigid-flexible coupling adhesive unit was established. Meanwhile, combined with the experimental test, the effect of the coupling mechanism of the rigid-flexible structure on the interfacial stress and the final peeling force during the compression and peeling process of the adhesive unit was studied. The research found that the length of the adhesive unit *L* has no apparent effect on the normal peel force of the system within a specific range, and the normal peeling force increases linearly with the increase in the compression force *P*; while the influence of the inclination angle *θ*_0_ of the adhesive unit and the thickness of the rigid backing layer *h*_b_ on the final normal peeling force of the system presents nonlinear characteristics, when the inclination angle *θ*_0_ of the adhesive unit is 5°, and the thickness of the rigid backing layer *h*_b_ is 0.2 mm or 0.3 mm, the normal peel force and the ratio of adhesion force to preload the system reaches its maximum value. Compared with the flexible adhesive unit, the compressed zone formed by the rigid-flexible coupling adhesive unit during the same compression process increased by 6.7 times, while under the same peeling force, the peel zone increased by 8 times, and the maximum normal tensile stress at the peeling end decreased by 20 times. The rigid-flexible coupling mechanics improves the uniformity of the contact stress during the compression and peeling process. The research results provide guidelines for the design of the rigid-flexible coupling adhesive unit, further providing the end effector of the bionic wall-climbing robot with a rigid-flexible coupled bionic design.

## 1. Introduction

There have been numerous morphological and adhesive force studies on biological dry adhesion mechanisms and adhesion systems [1,2], and the development of micro and nano processing technologies has made it possible to develop biomimetic dry adhesives comparable to biological adhesion, with high surface adaptability [3,4,5] and controllable adhesion [6,7]. However, the performance of existing bionic adhesive systems still lags far behind that of bioinspired adhesive systems, which inspired us to design biomimetic support systems for dry adhesives.

The attachment and detachment behavior of an adherent animal toe is the primary method of its interaction with the external environment. As a constituent part of the organism, the adherent terminal almost always exhibits a rigid-flexible coupling framework [8]. Taking the toe structure of the *Gekko gecko* in nature as an example, it typically consists of a microscopic setae (~3 GPa), amesoscopic lamella structure, muscle tissue (~3 MPa), and macroscopic phalangeal structures (~5 GPa) [9,10]. In order to reveal the excellent performance of the gecko adhesion system, researchers have developed various attachment and detachment models to understand the adhesion, friction, and peeling processes at the microscopic level [11,12]. Tian et al. [13] studied the rigid-flexible coupling system composed of setae and lamella structures in the *Gekko gecko*’s adhesive system, and the study showed that a proper selection of the stiffness of the flexible lamella structure could allow the adhesive system to maintain the adhesion state under a wide range of deformation. However, the structure–function relationship of the rigid-flexible coupling adhesive system remains a pressing issue in understanding biological adhesion systems and bionic adhesion design. How the coupling mechanism of the rigid-flexible structure affects the stress distribution at the contact interface of the adhesion system, avoiding stress concentration and thus, improving the loading capacity of the adhesive system, is still unknown.

The study of attachment and detachment behavior mainly focuses on the independent study of the formation and delamination process of the adhesive interface [14], such as the adhesively bonded joint [15], and there are few studies on the interaction between the formation and delamination of the adhesive interface. For applications such as wall climbing robots [16,17,18,19] or adhesive grippers [20,21,22], the end effector needs to adhere and peel off multiple times [23]; thus, the attachment and detachment process need to be studied systematically. Through traditional linear fracture mechanics and interfacial adhesion studies, the structural and mechanical characteristics of the peeling process have been well interpreted. The peeling characteristics of pressure-sensitive adhesive tapes (PSA) [24], peel joints [25], and some dry adhesion organisms [26] have been revealed and successfully applied to paint and coating technology and transfer printing [27,28]. The adhesive interface strength between the biomimetic dry adhesive material and a specific target surface is achieved by contact compression, and the adhesive interface strength generally depends on the (normal or tangential) compression [29,30]. The loading capacity of dry adhesive systems depends on the compression and peeling process, but a comprehensive study of both is still lacking.

In this paper, a contact mechanics model of adhesive units with rigid-flexible coupling characteristics during compression and peeling was established; further, the interface strength and peeling force of rigid-flexible coupling adhesive units with different geometric parameters (length and rigid layer thickness) and under different preloads were investigated by numerical analysis and experiments. This study can help to better understand organisms’ rigid-flexible coupling adhesive systems and reveal the excellent mechanical properties of the rigid-flexible coupling adhesive units.

## 2. Modeling and Numerical Analysis of the Rigid-Flexible Coupling Adhesive Unit

**Figure 1:**
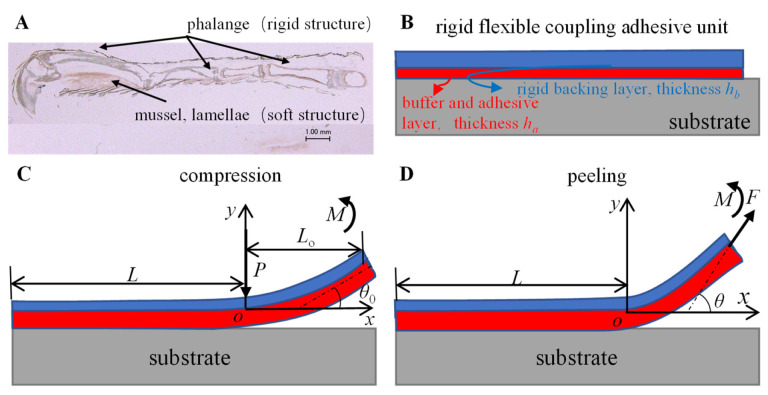
(**A**) Rigid-flexible coupling biological structure of gecko toes; (**B**) geometric model and mechanical analysis of rigid-flexible coupling adhesive unit during the (**C**) compression and (**D**) peeling process.

Morphological studies on the toe of the *Gekko* *gecko* [10] have revealed its rigid-flexible coupling biological mechanism (Figure 1A). A bioinspired rigid-flexible coupling adhesive unit (Figure 1B) was proposed in this paper. In order to systematically study the effect of its rigid-flexible coupling characteristics on the capture performance, a mechanical model of the adhesive unit during the compression and peeling process was established as follows.

### 2.1. Mechanical Model

The geometric model of the rigid-flexible adhesive unit is shown in Figure 1B. The adhesive unit consists of a rigid backing layer (RBL), with a thickness of *h*_b_, and a flexible buffer and adhesive layer (BAL), with a thickness of *h*_a_; the length of the entire adhesive unit is *L* and the width is *b*. It is assumed that both the RBL and the BAL satisfy the linear elasticity assumption, with elastic moduli *E*_b_ and *E*_a_, respectively, and the shear modulus of BAL is *μ*. During the compression process (Figure 1C), the normal preload *P* and moment *M* act on the right end of the contact area and the RBL, respectively. The length of the delamination region is *L*_0_, and the rotation angle of the right end is *θ*_0_. During the peeling process (Figure 1D), the peeling force *P* acts on the right end of the RBL at an angle of *θ* with the substrate. To facilitate the mechanical modeling, an *xoy* coordinate system was established, where the coordinate origin o is at the same horizontal position as the peeling front of the system and at the same vertical position as the undeformed BAL; the *x*-axis is parallel to the substrate to the right and the *y*-axis is perpendicular to the base upwards.

Due to the existence of minor normal and tangential strains of the adhesive unit during the compression and peeling process, the small deformation hypothesis is satisfied. Based on the Kaelble model [31], the equation of the normal deformation of the structure is

(1) $$\frac{d^{4}y}{dx^{4}}+4\beta^{4}y=0$$

where $\beta ={\left[3E_{a}/\left(E_{b}h_{b}{}^{3}+E_{a}h_{a}{}^{3}\right)h_{a}\right]}^{\frac{1}{4}}$, when *x* is in the interval [−*L*,0], *y* represents the normal deformation of the BAL. The general solution to this equation is

(2) $$y=e^{-\beta x}\left(C_{1}\cos\beta x+C_{2}\sin\beta x\right)+e^{\beta x}\left(C_{3}\cos\beta x+C_{4}\sin\beta x\right)$$

where C*_i_* (*i* = 1,2,3,4) are constants related to the normal boundary condition of the system.

The tangential deformation equation is

(3) $$\frac{d^{2}\gamma }{dx^{2}}-\alpha^{2}\gamma =0$$

where $\alpha ={\left(\mu /E_{b}h_{a}h_{b}\right)}^{\frac{1}{2}}$, when *x* is in the interval [−*L*,0], *γ* represents the tangential deformation of the BAL. The general solution to this equation is

(4) $$\gamma {=D}_{1}e^{\alpha x}+D_{2}e^{-\alpha x}$$

where D_1_ and D_2_ are constants related to the tangential boundary condition of the system.

During the compression process (Figure 1C), the tangential deformation is ignored, as the angle of inclination *θ*_0_ is slight. Considering the normal boundary conditions of the system as Equation (5).

(5) $$\begin{array}{l} \frac{bE_{a}}{h_{a}}\int_{-L}^{0}ydx=P\\ \frac{d^{2}y}{dx^{2}}\left|{}_{x=0}=\right.3\theta_{0}/L_{0}\\ \frac{d^{3}y}{dx^{3}}\left|{}_{x=-L}=0\right.\\ \frac{d^{2}y}{dx^{2}}\left|{}_{x=-L}=0\right. \end{array}$$

Substituting Equation (5) into Equation (2) and solving for the constant C*_i_* to obtain the normal displacement of the system $y_{\mathrm{load}}$ during compression, the normal contact stress of the system is

(6) $$\sigma_{\mathrm{load}}=\frac{E_{a}y_{\mathrm{load}}}{h_{a}}$$

During the peeling process (Figure 1D), considering its normal and tangential contact deformation, the normal boundary conditions of the system are

(7) $$\begin{array}{l} \frac{bE_{a}}{h_{a}}\int_{-L}^{0}ydx=F\sin\theta \\ E_{b}I\frac{d^{2}y}{dx^{2}}\left|{}_{x=0}=M_{C}\right.\\ \frac{d^{2}y}{dx^{2}}\left|{}_{x=-L}=0\right.\\ \frac{d^{3}y}{dx^{3}}\left|{}_{x=-L}=0\right. \end{array}$$

where the bending moment at *x* = 0 can be expressed as [32]

(8) $$M=\sqrt{2FE_{b}I_{b}\left(1-\cos\theta \right)}$$

Therefore, the bending moment *M*_C_ caused by the normal adhesive force can be expressed as

(9) $$M_{C}=\sqrt{2FE_{b}I_{b}\left(1-\cos\theta \right)}-\frac{1}{2}Fh_{b}\cos\theta$$

Considering the tangential boundary conditions as

(10) $$\begin{array}{l} b\mu \int_{-L}^{0}\gamma dx=F\cos\theta \\ \frac{d\gamma }{dx}\left|{}_{x=-L}=0\right. \end{array}$$

Substituting Equations (7) and (10) into Equations (2) and (4), respectively, the normal displacement $y_{\mathrm{peel}}$ and tangential strain $\gamma_{\mathrm{peel}}$ of the unit during the peeling process can be obtained, and the normal and tangential contact stresses are

(11) $$\begin{array}{l} \sigma_{\mathrm{peel}}=\frac{E_{a}y_{\mathrm{peel}}}{h_{a}}\\ \tau_{\mathrm{peel}}=\mu \gamma_{\mathrm{peel}} \end{array}$$

The influence of mechanical and geometric parameters of the rigid-flexible coupling adhesive unit on the contact stress characteristics during the compress and peeling process can be obtained by Equations (6) and (11). Then, the attachment and detachment behavior of the rigid-flexible coupling adhesive unit can be analyzed.

According to the research of Yuan et al. [33], the normal contact stress at the peeling front (*x* = 0) reaches the maximum value, while the maximum interfacial shear stress may appear at the loaded end, or a small distance from the loaded end, depending on the value of the peel angle. In this paper, the contact stress at the peeling front is used as the condition for the peeling behavior; then, it is necessary to study the stress failure mode and the traction versus separation law of the adhesive interface.

### 2.2. Traction Versus Separation Law

In order to predict the peeling force of the rigid-flexible coupling adhesive unit, the interfacial adhesive strength must be described. This paper uses the cohesive zone model (CZM) [34] to describe the interfacial traction versus the separation law.

**Figure 2:**
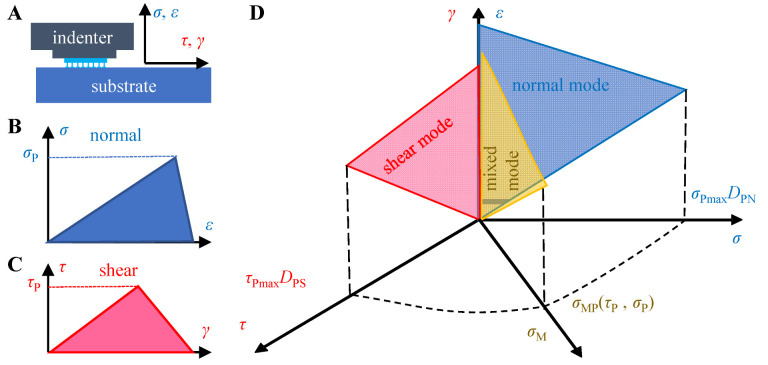
(**A**) Interfacial action during pull-off of the bioinspired adhesives; (**B**) normal and (**C**) tangential bilinear CZM models considering precompression strength; (**D**) two-dimensional coupled CZM model with precompression strength dependence.

A specific preload is essential to generate a certain adhesion and to form an adhesive interface. It was found that the interfacial adhesion was affected by the preload [35] and peeling forces acting in different directions (e.g., purely normal, purely tangential [36,37], and the coupling between the two [38], Figure 2A–C also shows different interfacial separation relationships.

In the test of the bioadhesive systems or bioinspired dry adhesives, the load-pull (LP) method or the load-drag-pull (LDP) method is usually used to characterize the adhesive performance [39,40]. Traditionally, the adhesive interface failure is accompanied by an energy balance between the external work, the adhesive energy of the nonfailure part, and the dissipated energy during crack evolution. In order to simplify the expression of dissipated energy, Dugdale et al. [41] took the lead in using CZM to describe the generation of interfacial crack regions and the formation of new interfaces. The CZM is also successfully applied to describe the breaking of composites and metal joints [42]. Here, we introduce CZM to simulate the interfacial failure between dry adhesives and rigid substrates. Assuming independent effects of the compress stress and pull-off directions on the traction versus separation relationship, a bilinear CZM [38,43] considering the compress stress can be obtained as Equation (12).

(12) $$\sqrt{{\left(\frac{\sigma_{P}}{\sigma_{\mathrm{Pmax}}D_{\mathrm{PN}}}\right)}^{2}+{\left(\frac{\tau_{P}}{\tau_{\mathrm{Pmax}}D_{\mathrm{PS}}}\right)}^{2}}=1$$

where $\sigma_{\mathrm{Pmax}}$ and $\tau_{\mathrm{Pmax}}$ are the normal and tangential pull-off stress under quasi-static conditions, respectively, and $D_{\mathrm{PS}}$ and $D_{\mathrm{PN}}$ are the influencing factors of precompression strength on tangential and normal interfacial strength, respectively. $\sigma_{\mathrm{Pmax}}$ and $\tau_{\mathrm{Pmax}}$ are saturation stress values in the normal and tangential directions, respectively. When $\sigma_{P}$ and $\tau_{P}$ satisfy Equation (12), the tensile force under the mixed mode is calculated as $\sigma_{\mathrm{MP}}=\sqrt{\sigma_{P}{}^{2}+\tau_{P}{}^{2}}$ (Figure 2D). The values in the bilinear CZM model considering the precompression strength are measured experimentally, and the results are given in the Supplementary Material. The first part of the Supplementary Material provides the experimental results and data fitting results [44] of the relationship between the adhesion strength and the precompression strength (Figure S1), and on this basis, the results of the precompression influence factors $D_{\mathrm{PS}}$ and $D_{\mathrm{PN}}$ were calculated; the second part provides the experimental results of the interface strength in different pull-off directions, and the saturation stress values in the normal and tangential directions ($\sigma_{\mathrm{Pmax}}$ and $\tau_{\mathrm{Pmax}}$) are obtained by ellipse fitting (Figure S2).

### 2.3. Mechanical Analysis Flow of Rigid-Flexible Coupling Adhesive Unit during the Compression and Peeling Process

**Figure 3:**
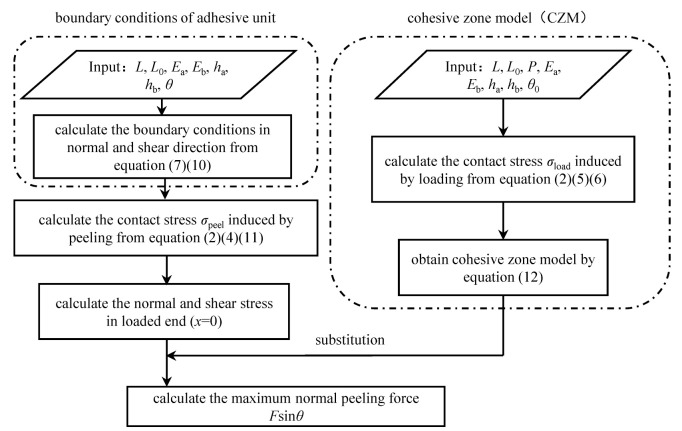
Mechanical analysis flow of rigid-flexible coupling adhesive unit during the compression and peeling process.

The peeling force between the adhesive unit and the substrate is determined by the interfacial adhesion stress formed during the compression process and the mechanical properties of the rigid-flexible coupling structure. According to the model of the rigid-flexible coupling adhesive unit, the model analysis flow of the adhesive unit in the compression and peeling process is established (Figure 3).

Firstly, the normal contact stress distribution of the system is calculated according to the normal deformation Equation (1) of the adhesive unit and the normal boundary condition Equation (5) during the compression process to obtain the maximum normal compressive stress; the coupling failure model of the adhesion interfaces is obtained by Equation (12). Combined with the normal and tangential deformation Equations (1) and (3) and boundary conditions Equations (7) and (10) of the adhesive unit in the peeling process, the normal peeling force *F*sin*θ* of the adhesive unit in the compression and peeling process under different working conditions is obtained.

## 3. Experiments and Methods

In order to verify the correctness of the model of the rigid-flexible coupling adhesive unit, adhesive units with different structural characteristics (different RBL thickness *h*_b_ and unit length *L*) were fabricated, and the theoretical and experimental studies under different load modes (different *P* and *θ*_0_) were carried out.

### 3.1. Simultaneous Test Method of the Quasi-Static Adhesion Mechanics and the Contact State

The contact force and geometry test technique were used to investigate the strength of the dry adhesive interface and the attachment-detachment mechanics of the rigid-flexible coupling adhesive unit. A simultaneous platform was built to obtain the quasi-static contact mechanics and the contact state.

**Figure 4:**
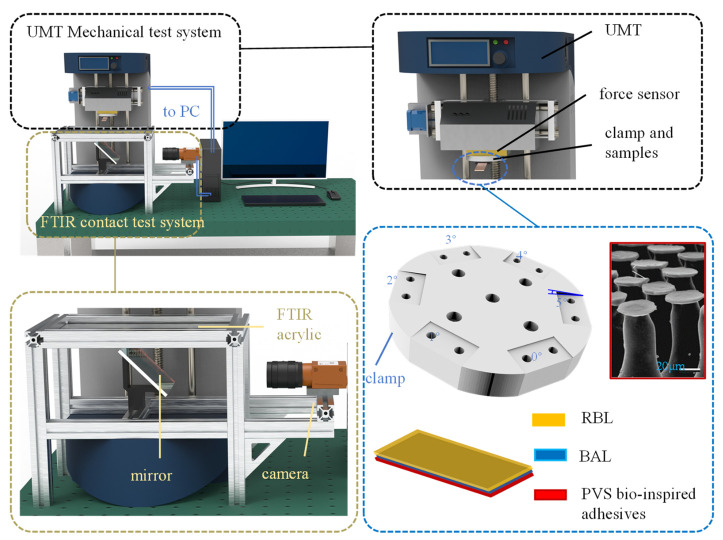
The synchronized testing platform for testing the adhesive contact state and mechanics.

The platform for synchronously testing adhesive contact state and mechanics (Figure 4) is mainly composed of the UMT (Universal Mechanical Tester system, Bruker Nano Inc., San Jose, CA, USA) mechanical test system and the FTIR (frustrated total reflection) [45,46] contact state test system. 

The contact image and mechanical results are synchronized on the PC. The UMT mechanical test system uses a two-dimensional force sensor (±50 N, 1000 Hz) to test the normal and tangential forces during the contact between the sample and the transparent acrylic plate. The FTIR contact test system consists of an FTIR acrylic (200 mm × 200 mm × 10 mm) with LED strips around the perimeter, a mirror placed under the acrylic at an angle of 45°, and a high-speed camera (Ispeed 3, Olympus, 300 Hz). The bioinspired adhesive used in this paper is a PVS surface with mushroom-shaped microstructures [5], and a flat indenter fixture is designed to carry the adhesive material samples. In order to prevent the stress concentration caused by the excessive plane size, the size of the indenter is 10 mm × 10 mm, and the size of the PVS adhesive sample is 5 mm × 5 mm. In order to further explore the interfacial adhesion-desorption mechanism of the rigid-flexible coupling adhesive unit, a clamp for fixing adhesive units with six tilt angles (0°~5°) was designed.

### 3.2. Working Conditions and Data Processing for the Attachment-Detachment Test of the Adhesive Unit

**Figure 5:**
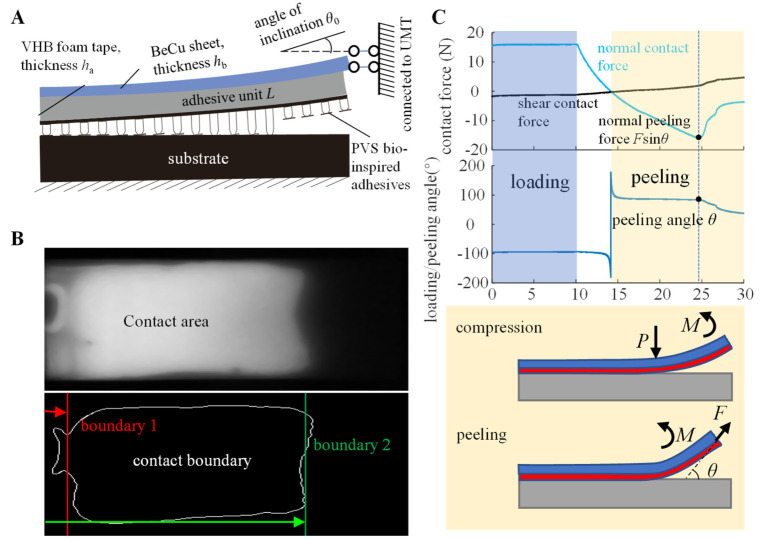
(**A**) Structural parameters and test conditions of the rigid-flexible coupling adhesive unit; (**B**) images of the contact area and boundary extraction acquired by the FTIR contact test system; (**C**) contact force and contact angle of the rigid-flexible coupling adhesive unit in the pre-press and peel stages acquired by the UMT mechanical test system.

As shown in Figure 5A, the adhesive unit includes a rigid, flexible, and adhesive layer, from top to bottom. The rigid layer is a beryllium copper sheet (Sheng Jili, Inc, Shenzhen, China) with a width of 17 mm, 30 to 50 mm in length, and 0.1 to 0.5 mm in thickness. The flexible layer is a 3 mm thick acrylic foam tape (3M™ VHB™ Tape 4959) bonded to the bottom of the rigid layer. The adhesive layer is a PVS bionic adhesive with mushroom-shaped microstructures bonded to the flexible layer’s bottom. The specific geometric parameters and mechanical properties of materials are shown in Table 1.

Since there are many experimental variables, we experimented with a simple control-variable approach, that is, when one variable is changed, the other variables remain unchanged. In order to maintain consistency and reduce errors, the loading and unloading speeds of the normal displacement during the test are both 0.1 mm/s. The specific test conditions are shown in Table 2.

We segmented videos captured by the high-speed camera into frames, then converted frames into grayscale images and obtained the contact area shown in Figure 5B through binary conversion. We extracted lengthwise contact boundaries 1 and 2 and measured the distance from each boundary to the free end. The contact ratio is defined as the ratio of the boundary displacement to the element length. The increase in the contact ratio means that the boundary moves to the right, and vice versa, the boundary moves to the left. The UMT mechanical testing system obtained the normal force and tangential force during the contact, and the normal peeling force *F*sin*θ* (maximum negative normal force) and the peeling angle *θ* were extracted simultaneously (Figure 5C). All tests were performed *n* = 5 times, and the results are expressed as the mean ± sd.

## 4. Result

### 4.1. Compression State of the Adhesive Unit

Based on van der Waals force, the adhesive unit forms an attraction between the PVS adhesive with mushroom-shaped microstructures and the target substrate. The normal and tangential adhesion forces are closely related to the initial contact state between the adhesive and the substrate. Therefore, the analysis of the compression state of the adhesive unit under different working conditions is the premise for studying its mechanical properties during the compression process.

**Figure 6:**
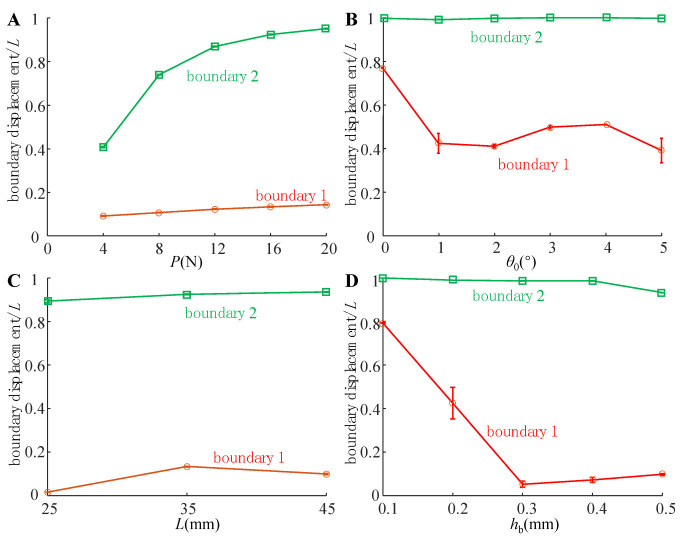
Boundary displacement of the adhesive unit for different (**A**) compression force *P*, (**B**) angle of inclination *θ*_0_, (**C**) unit length *L*, (**D**) and thickness of RBL *h*_b_.

As shown in Figure 6, the compression states of the adhesive unit at different preload *P* (condition 1), different inclined angle *θ*_0_ (working condition 2), different length *L* (working condition 3) and different rigid layer thickness *h*_b_ (working condition 4) were statistically analyzed. The experimental results show that boundary 2 rapidly moves to the right with the increase in *P*, and tends to the saturation value when *P* reaches 16N. However, the effects of *θ*_0_, *L*, and *h*_b_ on boundary 2 are not apparent. With the increase in *h*_b_, the adhesion boundary 1 rapidly moves to the left so that the actual adhesion area increases quickly, reaching the leftmost side at *h*_b_ = 0.3 mm, then moving slowly to the right. With the increase in *θ*_0_, boundary 1 tends to move to the left gradually, and the actual adhesion area is also increasing. However, the changes in *P* and *L* had no significant effect on boundary 1.

The strength of the adhesive interfaces depends on the preload stress. Although adhesion boundaries are exact, different preload stress may lead to a significant difference in the adhesive unit’s adhesion force (peeling force). However, there is currently no effective test method to obtain the interfacial contact stress of the adhesive unit when preloaded. Here, we further assume that the adhesive unit is in contact with the substrate over the entire length and use the numerical solution method in Section 2 to obtain characteristics of the stress distribution when the adhesive unit is in contact with the substrate under different working conditions.

**Figure 7:**
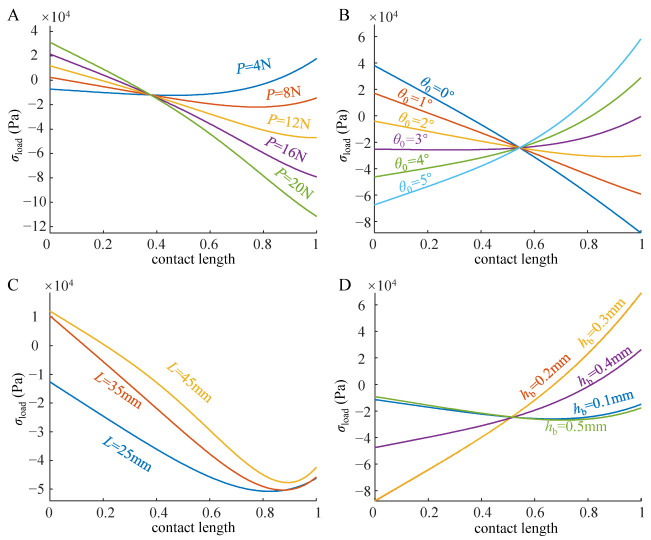
Numerical simulation results of the normal compression strengths at the adhesive interfaces for different (**A**) compression forces *P*, (**B**) angle of inclination *θ*_0_, (**C**) unit length *L*, and (**D**) thickness of RBL *h*_b_. The contact length is used as the horizontal coordinate, where a value of 0 for the contact length indicates the position of the free end of the adhesive unit, while a value of 1 for the contact length indicates the position near the solid support end of the adhesive unit.

When a slight preload (*P* = 4N) acts on the adhesive unit, the preload stress of the free end of the adhesive unit initially appears as compressive stress, while that of the fixed end appears as tensile stress. With the increase in *P*, the preload stress of the free end gradually decreases and turns into tensile stress (*P* > 16N), while that of the fixed end rapidly turns into compressive stress (*P* > 8N) and continues to increase (Figure 7A).

When *θ*_0_ increases, the preload stress of the free end of the adhesive unit gradually switches from tensile stress (*θ*_0_ < 2°) to compressive stress (*θ*_0_ > 2°), while that of the fixed end decreases continuously and switches to tensile stress at *θ*_0_ > 4° (Figure 7B). The increase in the tensile stress at the fixed end predicts the movement of the adhesion boundary 2 to the right, while the increase in the compressive stress at the free end predicts the movement of the adhesion boundary 1 to the left, which is consistent with the trend of the experimental results (Figure 6B).

With the increase in *h*_b_, the free end of the adhesive unit always maintains the compressive contact state. The compressive stress first increases, and then decreases, reaching the maximum value in the interval of 0.2mm < *h*_b_ < 0.3 mm. When *h*_b_ is small (*h*_b_ = 0.1 mm) or large (*h*_b_ = 0.5 mm), the preload stress of the fixed end appears as compressive stress, and when it is 0.2 mm < *h*_b_ < 0.4 mm, it appears as tensile stress (Figure 7D). The change in *L* does not significantly change the preload stress distribution (Figure 7C) and displacements of boundaries 1 and 2 (Figure 6C).

### 4.2. Peeling Behavior of the Adhesive Unit

The failure of adhesive interfaces accompanies the process of the adhesive unit peeling from the substrate. According to Section 2, the two most important factors affecting the failure of the adhesive interfaces are the initial preload stress and the peeling angle *θ*. Although the attachment-detachment direction of the adhesive units is perpendicular to the substrate in each working condition, the tangential force must accompany the peeling process, so the final peeling angle may not be equal to 90°. The normal and tangential forces measured during tests were analyzed, and the peeling angles *θ* during the traction-separation process of the adhesive unit under each working condition were obtained, as shown in Table 3. The *θ* decreases with the increase in *P*, and increases with the increase in *h*_b_. At the same time, *L* and *θ*_0_ have no significant effect on the final peeling angle *θ*. When *h*_b_ is small (*h*_b_ ≤ 0.2 mm), *θ* is less than 90°, and when *h*_b_ is large (*h*_b_ > 0.2 mm), *θ* is greater than 90°.

**Figure 8:**
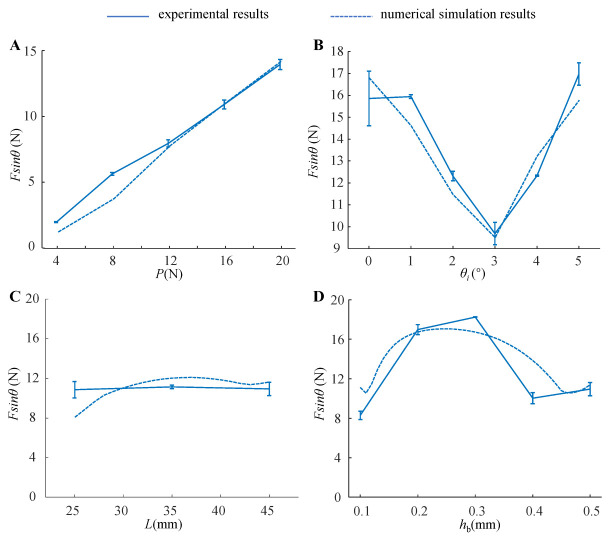
The normal peeling force *F*sin*θ* of the adhesive unit for different (**A**) compression force *P*, (**B**) angle of inclination *θ*_0_, (**C**) unit length *L*, and (**D**) thickness of RBL *h*_b_.

The numerical model analysis and experimental results of the normal peeling force between the adhesive unit and the substrate under different working conditions are shown in Figure 8. With the increase in *P* (Figure 8A), the normal peeling force increases linearly, and the ratio of adhesion force to preload (the ratio of the normal adhesive force *F*sin*θ* to the preload *P*) of the entire system under this condition is about 0.7. With the increase in the inclined angle *θ*_0_ (Figure 8B), *F*sin*θ* tends to decrease first, and increase afterward. When *θ*_0_ = 3°, *F*sin*θ* reaches a minimum value of 10N, and the ratio of adhesion force to preload is about 0.625. When *θ*_0_ = 0° or *θ*_0_ = 5°, *F*sin*θ* can exceed 16N, and the ratio of adhesion force to preload can be greater than 1 at this time. The length *L* of the adhesive unit has no significant effect on *F*sin*θ* (Figure 8C), and the ratio of adhesion force to preload is about 0.7. As the thickness *h*_b_ of the rigid layer increases, *F*sin*θ* increases and then decreases (Figure 8D); when *h*_b_ is selected to be 0.2 mm or 0.3 mm, the ratio of adhesion force to preload can be greater than 1.

## 5. Discussion

The mechanical modeling analysis and experimental test of the peeling performance of the adhesive unit reveal how the physical properties and geometrical structure of each component of the adhesive unit affect the final peeling force, which is the basis for realizing the complete application of the adhesive unit in various fields (such as wall-climbing robots and flexible adhesive graspers). Based on the previous modeling analysis and experimental results, the selection of the adhesive unit and mechanical parameters is studied to obtain a better adhesion performance of the adhesive system to inspire the establishment of the design criteria of the adhesive unit.

### 5.1. Comparison between Rigid-Flexible Coupling Adhesive Unit and Flexible Adhesive Unit

Based on the peeling energy for a single flexible adhesive unit, the Kendall model [25] can reasonably predict the peeling force at various peeling angles. However, the Kendall model does not consider the effect of bending stiffness on the peeling behavior, and the peeling behavior only occurs at the front end of the peel zone.

The PZ model [47] differs from the Kendall equation by considering the peeling zone; the length of the peel zone increases as the peel angle reduces. Hong Yuan et al. [33] found that the normal and tangential interfacial stress is concentrated near the loading end through the theoretical study of the thin plate structure peeling from the rigid substrate surface obliquely. In fact, according to the force balance principle, the forces of various adhesive units in the process of compression and peeling are distributed in the contact interfaces in a certain way, and this distribution determines the formation and delamination of the adhesion interface, which in turn determines the attachment-detachment mechanical behavior of the adhesive unit.

**Figure 9:**
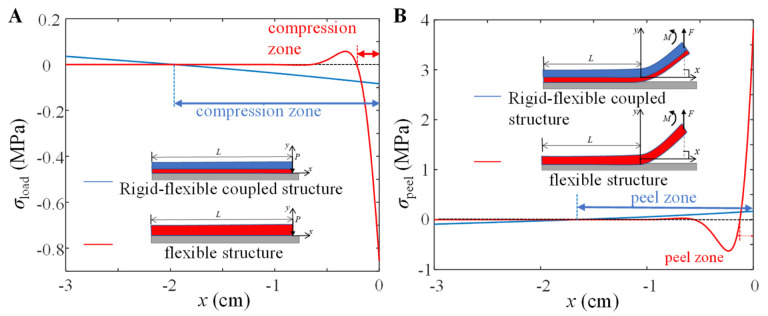
Numerical simulation results of the normal stress distribution of the rigid-flexible coupling adhesive unit and the flexible adhesive unit during the (**A**) compression process and (**B**) peeling process. The geometric parameters are taken as *h*_b_ = 0.2 mm, *h*_a_ = 3.4 mm, and *L* = 30 mm; the modulus of elasticity of the flexible adhesion unit is taken as 3 MPa, the normal force *F* = 10 N, and the peeling angle *θ* = 90°.

In order to reveal the excellent adhesion-desorption mechanical properties of the rigid-flexible coupling adhesive unit, this paper uses a single flexible adhesive unit as a comparison to study the stress distribution in the peeling/adhesive area and the final normal peeling force of these two adhesive units using the model. The normal contact stress distribution of these two adhesive units during the compression process is shown in Figure 9A. The compression zone of the flexible adhesive unit is only 1/10 of its length and concentrated near the loading end, while that of the rigid-flexible coupling adhesive unit is 2/3 of its length. Compared with the flexible adhesive unit, the rigid-flexible coupling adhesive unit has a more uniform stress distribution during the compression process and has a larger adhesive contact zone.

It is assumed that these two adhesive units are both in contact with the substrate and are fully preloaded. According to the analysis process in Figure 3, under the normal peeling force *F* = 10 N, the stress distribution of the adhesion interface is shown in Figure 9B. Under the normal force load, the peeling zone of the flexible adhesive unit is only about 1/15 of its length, and the maximum normal tensile stress at the peeling end is about 4 MPa, while the peeling zone of the rigid-flexible adhesive is more than 1/2 of its length, and the maximum normal tensile stress at the peeled end is only 0.2 MPa. Compared with the flexible adhesive unit, the rigid-flexible coupling adhesive unit has a larger peeling area, and the normal tensile stress at the peeling end is much smaller.

For the prediction of the peeling force of flexible adhesive units, the classic Kendall model [25] is introduced in this paper for comparison, and the expression for the peeling force is as follows.

(13) $$F=E_{a}h_{a}b\left(\cos\theta -1+\sqrt{{\left(1-\cos\theta \right)}^{2}+\frac{2G_{C}}{E_{a}h_{a}}}\right)$$

where *G*_C_ refers to the adhesion energy, which, according to the CZM model, is related to the peeling angle and compression force, and this can be expressed as

(14) $$G_{C}=\frac{\sigma_{P}{}^{2}}{2E_{a}}+\frac{\tau_{P}{}^{2}}{2\mu }$$

**Figure 10:**
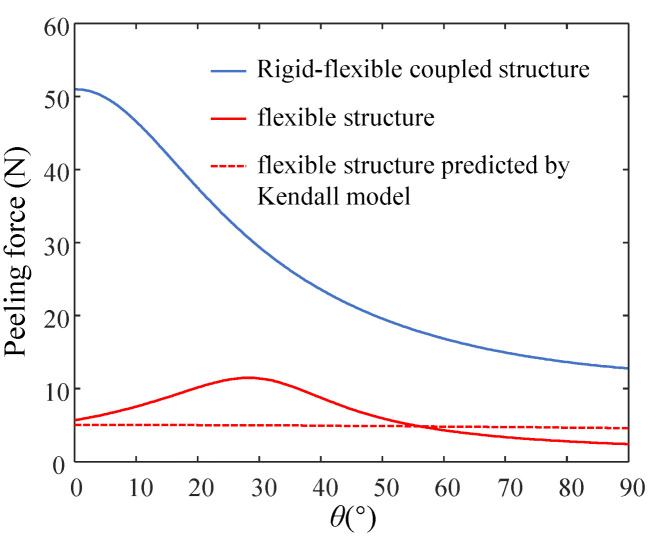
Variation of the peeling force with the peel angle for the rigid-flexible coupling adhesive unit and the flexible adhesive unit.

The peeling force of these two adhesive units under different peeling angles is shown in Figure 10. The peeling force of the rigid-flexible coupling adhesive unit decreases with the increase in the peeling angle, while the peeling force of the flexible adhesive unit increases first and then decreases with the increase in the peeling angle. Compared with the flexible adhesive unit, the rigid-flexible coupling adhesive unit has a more significant peeling force at each peeling angle; the peeling force predicted by the Kendall model is similar to the model proposed in this paper. The effect of the peeling force predicted by the Kendall model is roughly consistent with the results predicted by the model proposed in this paper.

### 5.2. Relationship between the Structural Characteristics and the Normal Loading Capability

Based on the force analysis of the rigid-flexible coupling adhesive unit during the compression and peeling process (Figure 1B,C), it can be seen that there are two main factors affecting the normal loading capability of the adhesion unit: one is the strength of the adhesion interface formed through the compression process, and the other is the coupling effect between the superstructure and the interface adhesion during the peeling process. We studied the adhesive unit with different structural characteristics (different RBL thickness *h*_b_ and unit length *L*) using theoretical models and experiments and conducted experimental studies of attachment-detachment under different loading modes (different compression force *P* and angle of inclination *θ*_0_). With the increase in compression force *P*, the compression zone gradually increases (Figure 6A), while the compressive stress value at the fixed end also increases accordingly (Figure 7A), and the final obtained normal peeling force increases linearly with the increase in compression force (Figure 8A). With the increase in the angle of inclination *θ*_0_, the contact stress at the adhesion boundary at the free end rapidly changes from tensile stress to compressive stress and gradually increases. In contrast, the contact stress at the fixed end gradually changes from compressive stress to tensile stress and increases (Figure 6B). The normal peeling force shows a “V-shaped” characteristic (Figure 8B). The maximum compressive stresses in the compression zone for different adhesive unit length *L* are about 0.05 MPa and occur at a compression zone length fraction of about 0.9 (Figure 7C), showing similar contact stress distribution and thus, similar final normal peeling forces (Figure 8C). As the thickness of the RBL hb increases, the compressive stress value at the free end tends to increase and then decrease. In contrast, the compressive stress value at the fixed end rapidly changes to tensile stress and finally switches to compressive stress (Figure 7D), resulting in an “inverted V-shaped” characteristic of the normal peeling force (Figure 8D).

**Figure 11:**
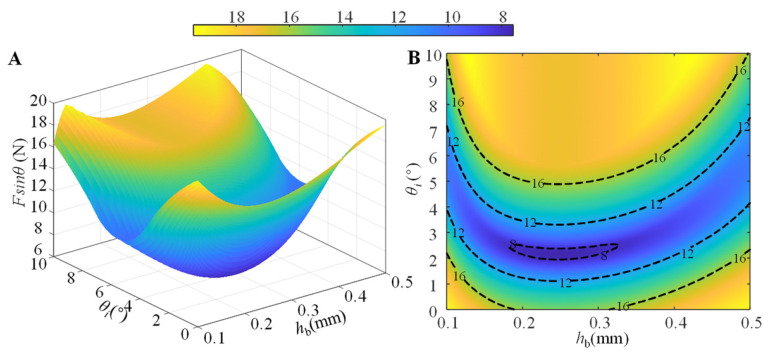
(**A**) Three-dimensional plot of the inclination angle *θ*_0_ and the thickness *h*_b_ of the RBL for the normal peeling force *F*sin*θ*; (**B**) the projection of the iso-*F*sin*θ* line on the *h*_b_-*θ*_0_ plane. The compression force *P* = 16 N is used in the simulation.

We further investigate the coupling effect of the angle of inclination *θ*_0_ and the thickness of the RBL *h*_b_ on the normal peeling force *F*sin*θ* by numerical simulation. The simulation results of the normal peeling force *P*sin*θ* at different inclination angle *θ*_0_ and thickness of RBL *h*_b_ are shown in Figure 11, where the orange area is the area where the adhesive unit can produce a higher adhesion force (normal peeling force is greater than 16 N); considering the compression force *P* of 16 N used in the simulation, the ratio of adhesion force to preload in this area is greater than 1. From Figure 11, it can be seen that when the inclination angle *θ*_0_ is smaller (*θ*_0_ ≤ 1°), the RBL thickness *h*_b_ can take the smaller (*h*_b_ = 0.1 mm) or larger (*h*_b_ ≥ 0.4 mm) values to achieve higher normal peeling force (greater than 16N) and the ratio of adhesion force to preload (greater than 1); when the inclination angle *θ*_0_ is 5°, the rigid layer thickness of the adhesion unit *h*_b_ takes 0.2 mm or 0.3 mm, and a maximum normal peeling force and the ratio of adhesion force to preload can also be obtained; although the increase in the inclination angle *θ*_0_ (especially when *θ*_0_ ≥ 6°) can increase the adhesion performance of the system to a large extent, the structure of the RBL in the adhesive unit also tends to produce plastic deformation, leading to the uncertainty in adhesion performance, which is not favorable to the reuse of the adhesive unit.

## 6. Conclusions

The rigid-flexible coupling mechanism of biological systems can improve the uniformity of the contact stress, which provides a solution for the design of bionic adhesion systems. In order to reveal the effect of the rigid-flexible coupling mechanism on the stress distribution at the contact interface of the adhesive system, a mechanical model of the rigid-flexible coupled adhesive unit during the compression and peeling process was developed in this study, and the interface adhesion strength was characterized by a bilinear coupled CZM model considering the pre-pressure effect. The results of the theoretical and experimental studies show that the variation of the length of the adhesive unit *L* has no obvious effect on the normal peeling force within a specific range, and the normal peeling force increases linearly with the increase in the compression force *P*; while the influence of the inclination angle *θ*_0_ of the adhesive unit and the thickness of the rigid backing layer *h*_b_ on the normal peeling force *F*sin*θ* exhibits nonlinear characteristics, the normal peeling force of the system can be maximized by optimizing the inclination angle *θ*_0_ and the thickness of the rigid backing layer *h*_b_. Compared with the flexible adhesive unit, the rigid-flexible coupling adhesive unit reduces stress concentration, significantly improving the compression zone during the compression process and the peeling zone during the peeling process; as a result, the normal tensile stress at the peeling end was dramatically reduced. The research results provide guidelines for the design of the rigid-flexible coupling adhesive unit, further providing the end effector of the bionic wall-climbing robot with a rigid-flexible coupled bionic design.

In future work, we will continue to improve the adhesive units’ surface adaptability (large curvature, flexibility, etc.) and dynamic response-ability (e.g., applications for climbing robots, flexible grippers, etc.). The existing regular, homogeneous structural units will be further extended to multi-layered, non-homogeneous, large flexible, and variable stiffness structural units. This will require the development of theoretical modeling and optimal design methods for multi-layered adhesive units, accurate testing of static and dynamic response capabilities, and consideration of the constraints of processing and material properties.

## Figures and Tables

**Table 1:** The specific geometric parameters and mechanical properties of the materials.

	RBL	BAL	PVS Adhesives
Length (mm)	30, 40, 50	30, 40, 50	30, 40, 50
Width (mm)	17	17	17
elastic module	128GPa	18 MPa	~3 MPa (bulk)
Poisson’s ratio	0.35	0.49	0.49
Thickness (mm)	0.1, 0.2, 0.3, 0.4, 0.5	3	0.4

**Table 2:** Working conditions of the adhesive unit during compression and peeling.

Working Condition	Thickness of RBL *h*_b_ (mm)	Length of the Unit *L* (mm)	Angle of Inclination *θ*_0_ (°)	Compression Force *P* (N)
working condition 1 (different *P*)	0.5	35	5	4, 8, 12, 16, 20
working condition 2 (different *θ*_0_)	0.2	45	0, 1, 2, 3, 4, 5	16
working condition 3 (different *L*)	0.5	25, 35, 45	5	16
working condition 4 (different *h*_b_)	0.1, 0.2, 0.3, 0.4, 0.5	45	5	16

**Table 3:** Peeling angle *θ* of adhesive unit for different compression force *P*, angle of inclination *θ*_0_, unit length *L*, and thickness of RBL *h*_b_.

		Peeling Angle (°) (Mean ± sd)			Peeling Angle (°) (Mean ± sd)			Peeling Angle (°) (Mean ± sd)			Peeling Angle (°) (Mean ± sd)
Preload (N)	4	109.3 ± 0.7	angle of inclination (°)	0	82.2 ± 0.3	thickness of RBL (mm)	0.1	74.7 ± 0.1	adhesive unit length (mm)	25	91.5 ± 0.1
	8	103.8 ± 0.4		1	82.7 ± 0.4		0.2	87.4 ± 0.2		35	92.5 ± 0.1
	12	108.2 ± 0.8		2	89.4 ± 0.4		0.3	91.9 ± 0.4		45	93.8 ± 0.4
	16	106.7 ± 0.8		3	85.5 ± 1.0		0.4	102.4 ± 0.2			
	20	104.0 ± 0.4		4	84.7 ± 0.1		0.5	93.8 ± 0.4			
				5	87.4 ± 0.2						

## Data Availability

The data generated and/or analyzed during the current study are not publicly available for legal/ethical reasons, but are available from the corresponding author upon reasonable request.

## Supplementary Materials

The following supporting information can be downloaded at: https://www.mdpi.com/article/10.3390/biomimetics7030119/s1, Figure S1: (A) normal contact stress-displacement curves at different precompression strengths $\sigma_{\mathrm{Pre}}$; (B) relationship between normal pull-off strength $\sigma_{P}\;$and precompression strength $\sigma_{\mathrm{Pre}}$; (C) tangential contact stress-displacement curves at different precompression strengths $\sigma_{\mathrm{Pre}}$; (D) relationship between tangential pull-off strength $\tau_{P}\;$and precompression strength $\sigma_{\mathrm{Pre}}$.; Figure S2: Relationship between normal pull-off strength $\sigma_{P}$ and tangential pull-off strength $\tau_{P}$ during directional pull-off at the adhesion interface.

## References

[1] Autumn K., Niewiarowski P.H., Puthoff J.B. (2014). Gecko Adhesion as a Model System for Integrative Biology, Interdisciplinary Science, and Bioinspired Engineering. Annu. Rev. Ecol. Evol. Syst..

[2] Lemetti L., Tersteegen J., Sammaljarvi J., Aranko A.S., Linder M.B. (2021). Recombinant Spider Silk Protein and Delignified Wood Form a Strong Adhesive System. ACS Sustain. Chem. Eng..

[3] Hu H., Tian H., Li X., Shao J., Ding Y., Liu H., An N. (2014). Biomimetic Mushroom-Shaped Microfibers for Dry Adhesives by Electrically Induced Polymer Deformation. ACS Appl. Mater. Interfaces.

[4] Davies J., Haq S., Hawke T., Sargent J. (2009). A practical approach to the development of a synthetic Gecko tape. Int. J. Adhes. Adhes..

[5] Gorb S., Varenberg M., Peressadko A., Tuma J. (2007). Biomimetic mushroom-shaped fibrillar adhesive microstructure. J. R. Soc. Interface.

[6] Parness A., Soto D., Esparza N., Gravish N., Wilkinson M., Autumn K., Cutkosky M. (2009). A microfabricated wedge-shaped adhesive array displaying gecko-like dynamic adhesion, directionality and long lifetime. J. R. Soc. Interface.

[7] Tao D., Gao X., Lu H., Liu Z., Li Y., Tong H., Pesika N., Meng Y., Tian Y. (2017). Controllable Anisotropic Dry Adhesion in Vacuum: Gecko Inspired Wedged Surface Fabricated with Ultraprecision Diamond Cutting. Adv. Funct. Mater..

[8] Zhou L., Ren L., Chen Y., Niu S., Han Z., Ren L. (2021). Bio-Inspired Soft Grippers Based on Impactive Gripping. Adv. Sci..

[9] Autumn K., Majidi C., Groff R.E., Dittmore A., Fearing R. (2006). Effective elastic modulus of isolated gecko setal arrays. J. Exp. Biol..

[10] Russell A.P. (1975). A contribution to the functional analysis of the foot of the Tokay, *Gekko gecko* (Reptilia: Gekkonidae). J. Zool..

[11] Kwak J., Kim T. (2010). A review of adhesion and friction models for gecko feet. Int. J. Precis. Eng. Manuf..

[12] Zhou M., Pesika N., Zeng H., Tian Y., Israelachvili J. (2013). Recent advances in gecko adhesion and friction mechanisms and development of gecko-inspired dry adhesive surfaces. Friction.

[13] Tian Y., Wan J., Pesika N., Zhou M. (2013). Bridging nanocontacts to macroscale gecko adhesion by sliding soft lamellar skin supported setal array. Sci. Rep..

[14] Kendall K. (1978). Interfacial dislocations spontaneously created by peeling. (Adhesive joint strength). J. Phys. D Appl. Phys..

[15] Banea M.D., Da Silva L.F. (2009). Adhesively bonded joints in composite materials: An overview. Proc. Inst. Mech. Eng. Part L J. Mater. Des. Appl..

[16] Asbeck A., Dastoor S., Parness A., Fullerton L., Esparza N., Soto D., Heyneman B., Cutkosky M. Climbing rough vertical surfaces with hierarchical directional adhesion. Proceedings of the 2009 IEEE International Conference on Robotics and Automation.

[17] Ko H., Yi H., Jeong H.E. (2017). Wall and ceiling climbing quadruped robot with superior water repellency manufactured using 3D printing (UNIclimb). Int. J. Precis. Eng. Manuf. Technol..

[18] Kim S., Spenko M., Trujillo S., Heyneman B., Santos D., Cutkosky M. (2008). Smooth Vertical Surface Climbing with Directional Adhesion. IEEE Trans. Robot..

[19] Wang B., Xiong X., Duan J., Wang Z., Dai Z. (2021). Compliant Detachment of Wall-Climbing Robot Unaffected by Adhesion State. Appl. Sci..

[20] Jiang H., Hawkes E.W., Fuller C., Estrada M.A., Suresh S.A., Abcouwer N., Han A.K., Wang S., Ploch C.J., Parness A. (2017). A robotic device using gecko-inspired adhesives can grasp and manipulate large objects in microgravity. Sci. Robot..

[21] Song S., Drotlef D.-M., Majidi C., Sitti M. (2017). Controllable load sharing for soft adhesive interfaces on three-dimensional surfaces. Proc. Natl. Acad. Sci. USA.

[22] Ruotolo W., Brouwer D., Cutkosky M.R. (2021). From grasping to manipulation with gecko-inspired adhesives on a multifinger gripper. Sci. Robot..

[23] Federle W., LaBonte D. (2019). Dynamic biological adhesion: Mechanisms for controlling attachment during locomotion. Philos. Trans. R. Soc. B Biol. Sci..

[24] Zhou M., Tian Y., Pesika N., Zeng H., Wan J., Meng Y., Wen S. (2011). The Extended Peel Zone Model: Effect of Peeling Velocity. J. Adhes..

[25] Kendall K. (1975). Thin-film peeling-the elastic term. J. Phys. D Appl. Phys..

[26] Persson B.N.J., Gorb S. (2003). The effect of surface roughness on the adhesion of elastic plates with application to biological systems. J. Chem. Phys..

[27] Meitl M.A., Zhu Z., Kumar V., Lee K.J., Feng X., Huang Y.Y., Adesida I., Nuzzo R.G., Rogers J.A. (2005). Transfer printing by kinetic control of adhesion to an elastomeric stamp. Nat. Mater..

[28] Da Silva L.F.M., Öchsner A., Adams R.D. (2011). Handbook of Adhesion Technology.

[29] Greiner C., del Campo A., Arzt E. (2007). Adhesion of Bioinspired Micropatterned Surfaces: Effects of Pillar Radius, Aspect Ratio, and Preload. Langmuir.

[30] Jiao Y., Gorb S., Scherge M. (2000). Adhesion measured on the attachment pads of *Tettigonia viridissima* (Orthoptera, Insecta). J. Exp. Biol..

[31] Kaelble D.H. (1960). Theory and Analysis of Peel Adhesion: Bond Stresses and Distributions. Trans. Soc. Rheol..

[32] Zhang L., Wang J. (2009). A generalized cohesive zone model of the peel test for pressure-sensitive adhesives. Int. J. Adhes. Adhes..

[33] Yuan H., Chen J., Teng J., Lu X. (2007). Interfacial stress analysis of a thin plate bonded to a rigid substrate and subjected to inclined loading. Int. J. Solids Struct..

[34] Barenblatt G.I. (1962). The Mathematical Theory of Equilibrium Cracks in Brittle Fracture. Adv. Appl. Mech..

[35] Paretkar D., Kamperman M., Martina D., Zhao J., Creton C., Lindner A., Jagota A., Mcmeeking R., Arzt E. (2013). Preload-responsive adhesion: Effects of aspect ratio, tip shape and alignment. J. R. Soc. Interface.

[36] Lu X.Z., Teng J.G., Ye L.P., Jiang J.J. (2005). Bond–slip models for FRP sheets/plates bonded to concrete. Eng. Struct..

[37] Li G., Tan K.H., Fung T.C., Del Linz P. (2020). Mode I fracture characterisation of FRP-concrete interfaces under dynamic loading. Compos. Struct..

[38] Li G., Tan K.H., Fung T.C., Yu Q.J., May M. (2021). A coupled dynamic cohesive zone model for FRP-concrete mixed-mode separation. Compos. Struct..

[39] Wang Y., Lehmann S., Shao J., Sameoto D. (2017). Adhesion Circle: A New Approach To Better Characterize Directional Gecko-Inspired Dry Adhesives. ACS Appl. Mater. Interfaces.

[40] Li X., Tao D., Lu H., Bai P., Liu Z., Ma L., Meng Y., Tian Y. (2019). Recent developments in gecko-inspired dry adhesive surfaces from fabrication to application. Surf. Topogr. Metrol. Prop..

[41] Dugdale D.S. (1960). Yielding of steel sheets containing slits. J. Mech. Phys. Solids.

[42] Dimitri R., Trullo M., De Lorenzis L., Zavarise G. (2015). Coupled cohesive zone models for mixed-mode fracture: A comparative study. Eng. Fract. Mech..

[43] Jiang W.-G., Hallett S.R., Green B.G., Wisnom M.R. (2007). A concise interface constitutive law for analysis of delamination and splitting in composite materials and its application to scaled notched tensile specimens. Int. J. Numer. Methods Eng..

[44] Schargott M., Popov V.L., Gorb S. (2006). Spring model of biological attachment pads. J. Theor. Biol..

[45] Song Y., Dai Z., Wang Z., Full R.J. (2020). Role of multiple, adjustable toes in distributed control shown by sideways wall-running in geckos. Proc. R. Soc. B Boil. Sci..

[46] Eason E.V., Hawkes E.W., Windheim M., Christensen D.L., Libby T., Cutkosky M.R. (2015). Stress distribution and contact area measurements of a gecko toe using a high-resolution tactile sensor. Bioinspir. Biomim..

[47] Pesika N.S., Tian Y., Zhao B., Rosenberg K., Zeng H., McGuiggan P., Autumn K., Israelachvili J.N. (2007). Peel-Zone Model of Tape Peeling Based on the Gecko Adhesive System. J. Adhes..

