# Supplementary material for: Effect of the Structural Characteristics on Attachment-Detachment Mechanics of a Rigid-Flexible Coupling Adhesive Unit
Source: Biomimetics (Basel). 2022 Aug 26;7(3):119. doi: 10.3390/biomimetics7030119 (PMC9496190; doi:10.3390/biomimetics7030119)
Supplement: Supplementary file 1 [file biomimetics-07-00119-s001.zip › biomimetics-1872308-Supplementary material.pdf]

Supplementary material

1. Results of Traction Versus Separation Tests of Adhesive Interface Under Different Precompression

Schargott et al. [47] proposed a Spring model to describe the relationship between pre-pressure and pull-off force in bioadhesive adhesion systems, and Greiner et al. [29] applied it to the design and characterization of bioinspired adhesives. The model results were in good agreement with the experimental results. In this paper, the Spring model is also used to describe the relationship between the pull-off strength and the precompression strength of the adhesives (Figure S1), and the relationships between the normal pull-off strength σ_P, the tangential pull-off strength τ_P, and the precompression strength σ_{Pre} can be obtained by fitting the experimental data, as shown in Equations (S1) and (S2), respectively. When the pre-pressure strength reaches 0.114 MPa and 0.098MPa, the normal and tangential pull-off strength reaches saturation values of 0.218 MPa and 0.098 MPa, respectively.

$$\sigma_P = \begin{cases} 0.986\sqrt{\sigma_{Pre}} - \sigma_{Pre} & \text{when } \sigma_{Pre} < 0.114 \\ 0.218 & \text{when } \sigma_{Pre} \geq 0.114 \end{cases} \quad \text{(S1)}$$

$$\tau_P = \begin{cases} 0.624\sqrt{\sigma_{Pre}} - \sigma_{Pre} & \text{when } \sigma_{Pre} < 0.098 \\ 0.098 & \text{when } \sigma_{Pre} \geq 0.098 \end{cases} \quad \text{(S2)}$$

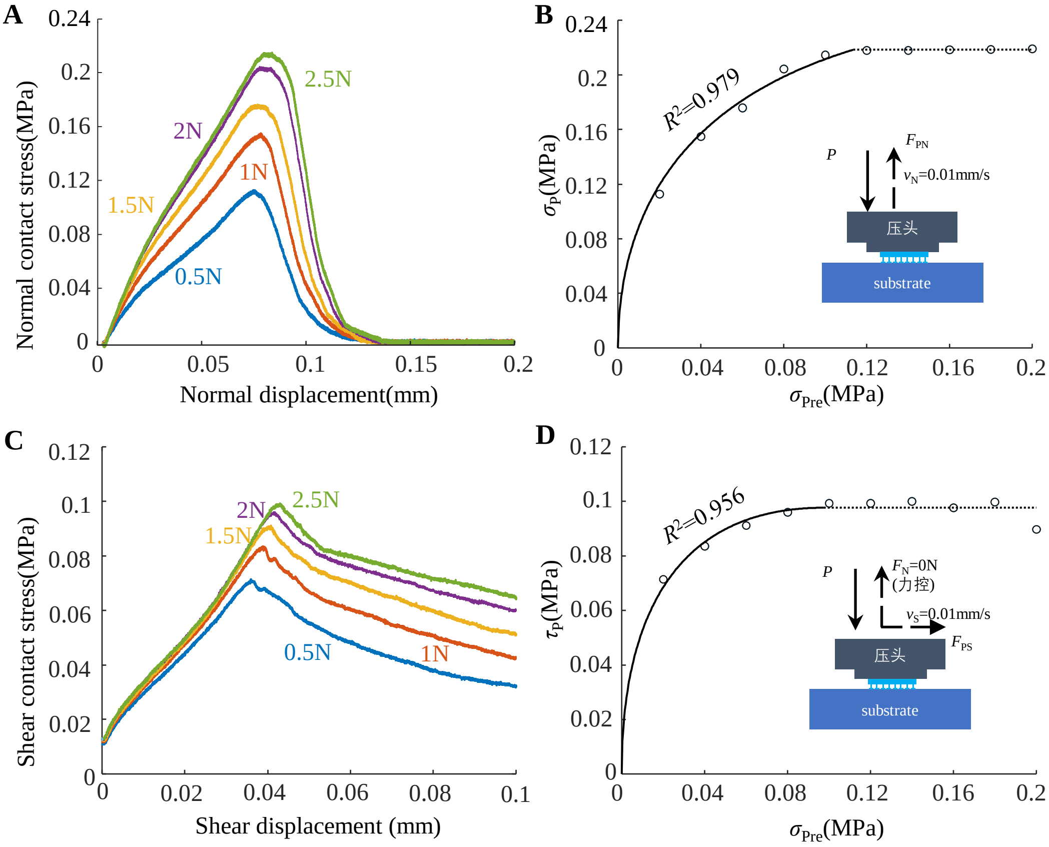

Figure S1. (**A**) normal contact stress-displacement curves at different precompression strengths σ_{Pre}; (**B**) relationship between normal pull-off strength σ_P and precompression strength σ_{Pre}; (**C**) tangential contact stress-displacement curves at different precompression strengths σ_{Pre}; (**D**) relationship between tangential pull-off strength τ_P and precompression strength σ_{Pre}.

The precompression influence factors D_{PS} and D_{PN} were introduced to describe the influence of tangential and normal precompression strength on the adhesion interface strength, respectively, defined as follows.

$$D_{PN} = \begin{cases} \dfrac{0.986\sqrt{\sigma_{Pre}} - \sigma_{Pre}}{0.218} & \text{when } \sigma_{Pre} < 0.114 \\ 1 & \text{when } \sigma_{Pre} \geq 0.114 \end{cases} \tag{S3}$$

$$D_{PS} = \begin{cases} \dfrac{0.624\sqrt{\sigma_{Pre}} - \sigma_{Pre}}{0.098} & \text{when } \sigma_{Pre} < 0.098 \\ 1 & \text{when } \sigma_{Pre} \geq 0.098 \end{cases} \tag{S4}$$

2. Results of Traction Versus Separation Tests of Adhesive Interface Under Different Precompression

Previous theoretical and experimental studies on the multi-axial stress damage criterion have shown that the normal and tangential stresses at the fracture damage interface approximately satisfy the elliptic equation [38,43]. In order to describe the traction versus separation relationship of the adhesive interface under coupled normal and tangential force, the elliptic equation is also introduced here.

$$\sqrt{\left(\frac{\sigma_P}{\sigma_{Pmax}}\right)^2 + \left(\frac{\tau_P}{\tau_{Pmax}}\right)^2} = 1 \tag{S5}$$

When the selected mixed loading mode, σ_P and τ_P satisfy Equation (S5), the value of the pull-off stress loaded along this direction is $\sqrt{\sigma_P{}^2 + \tau_P{}^2}$. As shown in Figure S2, the least-squares fitting of the test data yields $\sigma_{Pmax} = 0.18$MPa and $\tau_{Pmax} = 0.09$MPa, the physical significance of which is the pure normal and pure tangential pull-off strengths at the adhesion interface under quasi-static conditions (Figure S2).

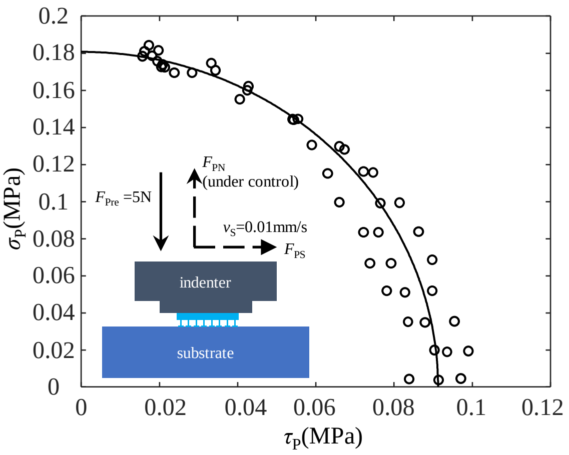

Figure S2. Relationship between normal pull-off strength σ_P and tangential pull-off strength τ_P during directional pull-off at the adhesion interface.
